# The Therapeutic Effects of DDP/CD44-shRNA Nanoliposomes in AMF on Ovarian Cancer

**DOI:** 10.3389/fonc.2022.811783

**Published:** 2022-03-25

**Authors:** Ting Guo, Yinxing Zhu, Miao Yue, Fujin Wang, Zhifeng Li, Mei Lin

**Affiliations:** ^1^Institute of Clinical Medicine, Taizhou People’s Hospital Affiliated to Nanjing University of Chinese Medicine, Taizhou, China; ^2^Clinical Laboratory, Taizhou People’s Hospital Affiliated to Nanjing University of Chinese Medicine, Taizhou, China

**Keywords:** PEG-MZF-NPs, magnetic fluid hyperthermia, ovarian cancer, DDP, CD44-shRNA

## Abstract

**Background:**

Globally, ovarian cancer is one of the most common gynecological malignant tumors, and the overall curative effect has been unsatisfactory for years. Exploring and investigating novel therapeutic strategy for ovarian cancer are an imperative need.

**Methods:**

Using manganese zinc ferrite nanoparticles (PEG-MZF-NPs) as gene transferring vector and drug delivery carrier, a new combinatorial regimen for the target treatment of ovarian cancer by integrating CD44-shRNA, DDP (cisplatin) and magnetic fluid hyperthermia (MFH) together was designed and investigated *in vivo* and *in vitro* in this study.

**Results:**

PEG-MZF-NPs/DDP/CD44-shRNA nanoliposomes were successfully prepared, and TEM detection indicated that they were 15–20 nm in diameter, with good magnetothermal effect in AMF, similar to the previously prepared PEG-MZF-NPs. Under the action of AMF, PEG-MZF-NPs/shRNA/DDP nanoliposomes effectively inhibited ovarian tumors’ growth, restrained the cancer cells’ proliferation and invasion, and promoted cell apoptosis. VEGF, survivin, BCL-2, and BCL-xl proteins significantly decreased, while caspase-3 and caspase-9 proteins markedly increased both *in vitro* and *in vivo*, far better than any of the individual therapies did. Moreover, no significant effects were found on bone marrow hematopoiesis and liver and kidney function of nude mice intervened by the combinatorial therapeutic regimen.

**Conclusion:**

In the present study, we developed PEG-MZF-NPs/DDP/CD44-shRNA magnetic nanoliposomes and inaugurated an integrated therapy through the synergistic effect of MFH, gene therapy, and chemotherapy, and it shows a satisfactory therapeutic effect on ovarian cancer *in vitro* and *in vivo*, much better than any single treatment regimen did, with no significant side effects. This study provides a new promising method for ovarian cancer treatment.

## Introduction

Ovarian cancer is one of main malignancies causing death in women worldwide (1). Currently, surgical resection is mainly used in the treatment of early ovarian cancer, while chemotherapy is used in advanced stages. In spite of good curative effect of surgery for early tumor, most patients have been in advanced stages when diagnosed because of the “silence” of ovarian cancer, and therefore, operative treatment is unfit for them. Chemotherapy possesses adverse effects, and patients could easily relapse for acquired drug resistance and metastasis (2). Thus, exploring a new efficient strategy for ovarian cancer treatment has been a hot topic scope of modern medical research.

Adhesion molecule CD44, a cell surface type 1 hyaluronic acid transmembrane glycoprotein receptor, is involved in the regulation of cell proliferation, cell differentiation, cell migration, angiogenesis, and inflammation (3). In tumor development, it is related to various metabolic pathways (4). It has been proven that CD44 is highly expressed in ovarian cancer, and its aberrant expression plays a crucial role in ovarian cancer occurrence, development, invasion, and metastasis (5). Other than that, CD44 is highly expressed in ovarian cancers’ drug-resistant cells and advanced epithelial tissues and has been considered a marker for poor ovarian cancer prognosis (5, 6). Hence, CD44 might be a conceivable target in ovarian cancer treatment. It was reported that blocking CD44 gene by RNA interference (RNAi) could significantly inhibit the growth of ovarian cancer cells and the formation of tumor blood vessels and reduce the recurrence and metastasis of tumors (2). CD44 gene knockout can increase the chemosensitivity of ovarian cancer cells to paclitaxel and inhibit tumor growth (7), and targeted inhibition of CD44 gene expression combined with chemotherapy drugs may be a more effective strategy for ovarian cancer treatment.

Honestly, as a modern treatment after traditional surgery, radiotherapy, and chemotherapy (8), gene therapy has an applicable prospect in cancer treatment. In recent years, copious tumor researchers have employed gene therapy in their investigations, among which RNAi has been proven efficacious in cancer therapy. However, efficient and stable gene transfer is pivotal in gene therapy.

With the development of nanotechnology, the use of nanoparticles as gene carriers has been widely studied (9). Magnetic nanoparticles can be directionally controlled under the action of the external magnetic field, exhibiting the unique characteristics of general nanoparticles and also sustain superparamagnetism, which can carry out efficient magnetic transfection (10). Tumor hyperthermia can also be achieved through magnetic nanoparticles under the action of an external magnetic field (11). This local and minimally invasive hyperthermia is designed to inhibit cancer cells’ growth and improve the effect of chemotherapy. Optimizing cancer cells’ drug delivery amalgamated with the MFH under the action of an alternating magnetic field (AMF) has become an intriguing research subject (12).

**Figure 1:**
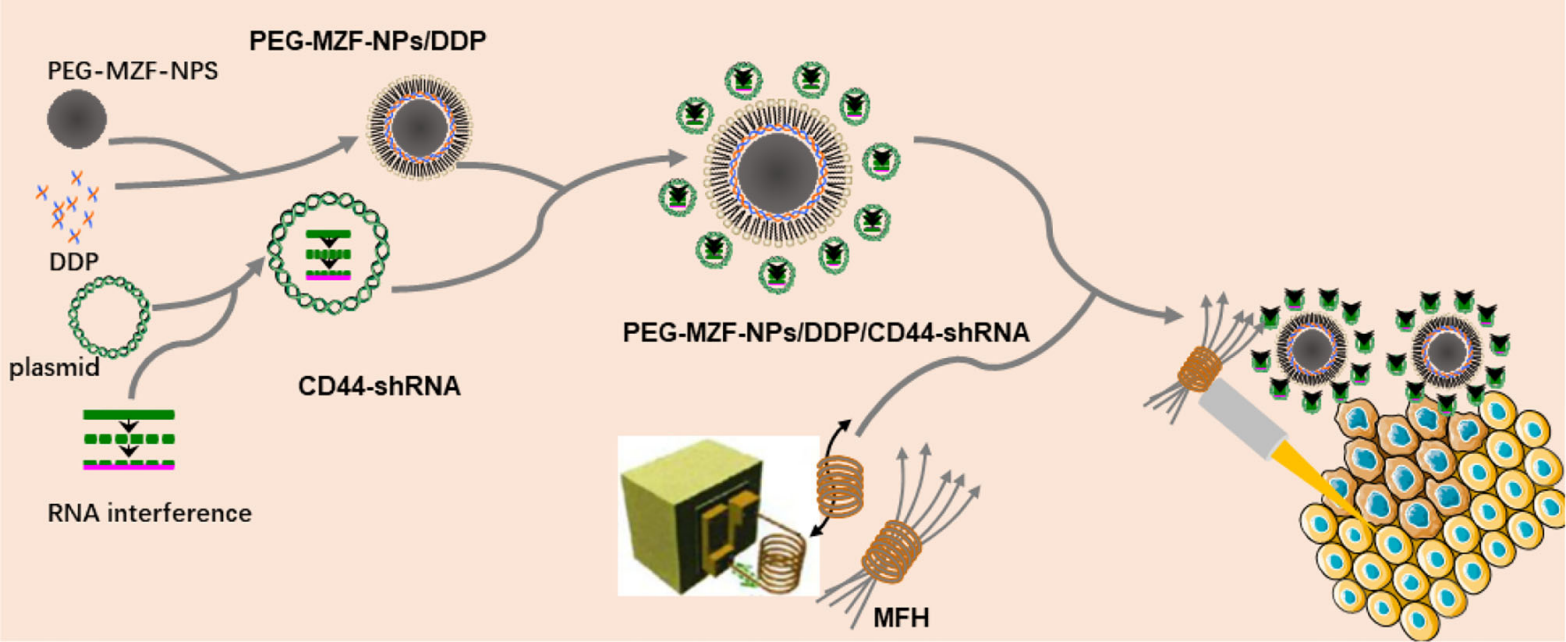
Schematic diagram of preparation of PEG-MZF-NPs/DDP/CD44-shRNA nanoliposomes and magnetic fluid hyperthermia therapy.

In the present study, DDP/CD44-shRNA nanoliposomes were prepared by using PEG-modified manganese zinc ferrite nanoparticles (PEG-MZF-NPs) previously developed as gene and drug carrier. Making use of PEG-MZF-NPs’ excellent magnetic response and effective temperature control, CD44-shRNA gene therapy, DDP chemotherapy, and magnetic thermotherapy were organically combined together to treat ovarian cancer *in vitro* and *in vivo* (**Figure 1**).

## Materials and Methods

### Materials

Cisplatin (DDP) was from Haosen Pharmaceutical Group Co., Ltd., Jiangsu, China. The total RNA extraction kit, reverse transcription kit, high-purity gel extraction kit, Western blotting test kit, and ECL test kit were all purchased from TaKaRa, Dalian, Liaoning, China. AnnexinV-FITC apoptosis kit was purchased from BD Company, BD Bioscience, San Jose, CA, USA. The secondary antibodies and the sheep anti-rabbit IgG were purchased from Sangon Biotech, Shanghai, China. Anti-VEGF, anti-survivin, anti-BCL-2, anti-BCL-xl, anti-cleaved caspase-3, and anti-cleaved caspase-9 antibodies were from Sigma, Newark, DE, USA. The rest of the reagents were from Invitrogen, MBI, Waltham, MA, USA.

### Preparation of PEG-MZF-NPs/DDP Nanoliposomes and Detection of the Physicochemical Properties

One hundred microliters (60 μg/ml) of PEG-MZF-NPs prepared previously was mixed with sufficient DDP (17 μg/ml) (13). Lecithin, cholesterol, and distilled water were added to the mixture and dispersed by ultrasound for 15 min and then filtered. The resulted filtrate encompassed PEG-MZF-NPs/DDP nanoliposomes. The hydrate particle size of PEG-MZF-NPs/DDP nanoliposome was detected by dynamic light scattering (DLS). The nanoliposome’s stability was tested at room temperature. Fourier transform infrared spectroscopy (FTIR) was used to analyze the alterations between PEG-MZF-NPs and PEG-MZF-NPs/DDP. We also determined the samples’ iron content using o-phenanthroline spectrophotometry. The heating effect of PEG-MZF-NPs/DDP nanoliposomes in AMF was examined as follows.

One milliliter of PEG-MZF-NPs/DDP nanoliposomes with Fe at 20, 40, 60, and 80 µg/ml was taken into a flat-bottomed test tube, respectively. Then, these tubes in turn were placed in an alternating magnetic induction instrument [SPG-10 (A)-11, Shenzhen, China] for 1 h, with a distance of 5 mm from the tube bottom to the hyperthermia-coil center. The output frequency was 235 kHz, and the output current was 35 A; the temperature was recorded every 5 min. Heating curves of PEG-MZF-NPs/DDP nanoliposomes with different concentrations were drawn by using temperature for the ordinate and time for the abscissa. Other than that, the magnetothermal effect of PEG-MZF-NPs/DDP nanoliposomes (Fe: 60 µg/ml) was examined when the output frequency was 235 kHz, and the output current was set to 15, 25, 35, and 45 A, respectively, and heating curves at different magnetic field intensity were drawn as the above. According to the heating curves, the applicable concentration of Fe and output current were selected in the later experiments.

### CD44-shRNA Plasmids’ Construction and Transfection

CD44-shRNA plasmids were synthesized according to the reference (14, 15) by Suzhou Jin Weizhi Co. Ltd, Jiangsu. The sequences of CD44-shRNA were as follows: shRNA-1, GGCAACTCCTAGTAGTACA; shRNA-2, GCGCAGATCGATTTGAATATA; and shRNA-NC, TTCTCCGAACGTGTCACGT. Ovarian cancer HO8910 cells were transfected with CD44-shRNA plasmids by PEG-MZF-NPs, and then, the expression of CD44 mRNA and protein was tested by real-time PCR (RT-PCR) and Western blot as follows.

PEG-MZF-NPs, CD44-shRNA, and negative control plasmids were dispersed and incubated in Iscove’s modified Dulbecco’s medium (IMDM) culture medium. After 30 min, PEG-MZF-NPs and CD44-shRNA at 40:1 ratio were mixed. HO8910 cells were cultured in serum-free IMDM containing the above mixture. Six hours later, the medium mixture was replaced with fresh IMDM.

After an additional 24 h, the cells were collected, and the CD44 mRNA expression was detected by SYBR GREEN PCR Master-Mix (RT-PCR, Applied Biosystem, Carlsbad, CA, USA). The PCR primer sequences used in the detection were sense-5murGGCACCCGATGTCCAGAA Murray 3 and antisense-5murcccctgaagtgctcccmur3 (Beyotime, Shanghai, China). The internal control glyceraldehyde 3-phosphate dehydrogenase (GAPDH) primer sequences were as follows: sense-5-catcatctttttgccccamur3 and antisense-5-TTAAAAGCAGCCCTGGTGACC-3 (sense-CGCACCCGCTCTATGTCCAGAMAUT, antisense-GGCACCCGCTATGTCCAGAA-3). The PCR protocol was as follows: 95°C cycle for 5 min, 43 cycles (95°C for 5 s, 60°C for 15 s, 72°C for 30 s).

Proteins were extracted using radioimmunoprecipitation assay (RIPA) buffer. The protein samples were separated by sodium dodecyl sulfate–polyacrylamide gel electrophoresis (SDS-PAGE) and then transferred to polyvinylidene fluoride membrane by sulfuric acid polyacrylamide gel electrophoresis. After transferring, the membranes were blocked in 5% skimmed milk at room temperature for 1 h, followed by incubation with the desired primary antibody overnight at 4°C. The next day, the membranes were incubated with a secondary antibody for 1 h and then subjected to protein visualization using enhanced chemiluminescence (Takara ECL substrate). BOXchemiXR5 imaging system was used in protein visualization.

### Preparation of PEG-MZF-NPs/DDP/CD44-shRNA Nanoliposomes

PEG-MZF-NPs/DDP/CD44-shRNA nanoliposomes were prepared by the thin film-ultrasonic method and high-speed stirring as follows: (1) PEG-MZF-NPs/DDP nanoliposomes initial preparation was mentioned above; (2) gelatin was dissolved in phosphate-buffered saline (PBS) buffer at 60°C in a water bath; and (3) solutions from steps 1 and 2 were mixed, followed by adding the above CD44-shRNA plasmids and stirring at 2,000 rpm until the formed film gravitated and hydrated. The supernatant and the unencapsulated nanomaterials at the bottom of the test tube were discarded by ultrasound and centrifugation. The resulted middle brownish aqueous suspension containing PEG-MZF-NPs/DDP/CD44-shRNA nanoliposomes were separated. Transmission electron microscopy (TEM) was used to observe the nanoliposomes’ morphology and particle size.

### Using PEG-MZF-NPs/DDP/CD44-shRNA Nanoliposomes Combined With MFH to Intervene HO8910 Cells *In Vitro*

HO8910 cells were purchased from the Shanghai Institute of Cell Research, Chinese Academy of Sciences (Shanghai, China). HO8910 cells were inoculated in IMEM culture medium (Sigma, US) containing 10% fetal bovine serum (GIBCO, US) and supplemented with streptomycin and penicillin (GIBCO Company). The cells at logarithmic growth phase were used for the involved experiments. The cells were divided into specific groups as follows: (1) negative control, (2) DDP, (3) DDP/CD44-shRNA, (4) PEG-MZF-NPs/MFH, (5) PEG-MZF-NPs/CD44-shRNA/MFH, (6) PEG-MZF-NPs/DDP/MFH, and (7) PEG-MZF-NPs/DDP/CD44-shRNA/MFH. All groups involved DDP contained the same volume of DDP. PEG-MZF-NPs were used in CD44-shRNA cell transfection in a mass ratio of 40:1. According to the above heating curves, the concentration of Fe at 60 µg/ml, the output frequency at 235 kHz, and the output current at 35A were selected. Groups 4–7 were placed on a high-frequency magnetic induction heating instrument [SPG-10 (A)-11, Shenzhen, China] for 1 h (4 kW, 235 kHz, 35 A), while the remaining groups were placed at room temperature for 1 h. All the above groups were routinely incubated in a saturated humidity incubator at 37°C and 5% CO_2_.

### The Assays for Cell Proliferation Inhibition *In Vitro*

After routinely incubated for 24 h, the cells from the above each group were seeded into 96-well plate, respectively, and then continued to be incubated for 24 h. After each well of all the plates were subjected to 20 μl MTT solution and routinely incubated for 4 h, 150 μl dimethyl sulfoxide (DMSO) was added into each well. The OD value was read at a wavelength of 500 nm. The cell proliferation inhibition rate was calculated using this formula (%) = [1 − (experimental group OD − blank OD)/(control group OD − blank OD)] × 100%.

### Cell Invasion Inhibition Analysis

Matrigel matrix glue was diluted using a serum-free medium and added to 24-well Transwell plates and incubated at 37°C. Some cells from the above each group incubated for 24 h were added to the superior compartment in the Transwell plates, followed by the addition of culture medium containing 10% fetal bovine serum (FBS) in the inferior chamber as chemotaxis. The non-invasive cells on the filter’s inner surface were washed off, and the outer surface was stained with crystal violet. The cell invasion rate was calculated following this formula (%) = cells’ count of the treatment group/cells’ count of untreated group × 100%.

### Cells’ Apoptosis Analysis

The remaining cells from each pre-established group incubated for 24 h were digested with trypsin and resuscitated with 1× binding buffer (100 μl), then transferred to the flow-cytometry tubes. Annexin-V and PI (5 μl) were added to each sample and incubated in room temperature and protected from light for 15 min. Binding buffer (1×) (400 μl) was added to each sample, and then, the cells’ apoptosis was analyzed by flow cytometry (BD, USA).

### *In Vivo* Analysis and Animal Modeling

Six-week-old female BALB/C nude mice, weighing 20–22 g, were purchased from (Shrek Experimental Animal Co., Shanghai, China). The animal experiment was approved by the Animal Protection Committee of Jiangsu Province (No. YXYLL-2021-63). All mice were kept and fed in the aseptic barrier system of the Medical College of Nantong University in Jiangsu Province, China. When the tumor-bearing mice were cultured for 1 week, the mice were randomly divided into seven groups as the same with the experiment *in vitro*. Each mice group were subjected to certain kind of injection, in which saline solution (0.5 ml/mice) was injected into the tumor in the group: (1) DDP (3mg/kg) was injected into the tumor of the group; (2) DDP (3 mg/kg) and CD44-shRNA (10 μg/mice) were injected into the tumor in the group; (3) the CD44-shRNA/PEG-MZF-NPs nanoliposomes (2 ml/kg; Fe, 60 μg/ml) were injected into the tumors in the group; (4) PEG-MZF-NPs (2 ml/kg; Fe, 60 μg/ml) was injected into the tumors of the group; (5) PEG-MZF-NPs/DDP nanoliposomes (10 ml/kg; Fe, 60 μg/ml) was injected into the tumors in the group; (6) the CD44-shRNA/DDP/PEG-MZF-NPs nanoliposomes (10 ml/kg; Fe, 60 μg/ml) were injected into the tumors in the group; and (7) 24 h later, tumor-bearing mice from groups 4–7 were anesthetized intraperitoneally using a 0.5% phenobarbital sodium solution (60 mg/kg). After complete anesthesia, the tumor-bearing mice were irradiated for 30 min using the high-frequency magnetic induction system (4 kW, 235 kHz, and 35 A) every 2 days in the first 2 weeks. The volume of each tumor was measured by color Doppler ultrasound method, and the changes in tumor growth were recorded for 6 weeks.

### Tumor Mass and Volume Inhibition Analysis

After being treated for 6 weeks, the nude mice were sacrificed, and the tumor tissues were stripped. The tumors’ mass and size were measured, and the tumor inhibition rate was calculated. The mass inhibition rate was calculated following this formula: 1 − tumor mass values from experimental group/tumor mass from the negative control group) × 100%. Similarly, the volume inhibition rate was calculated as (1 − tumor volume in the experimental group/tumor volume in the negative control group) × 100%.

### Histopathological and Biochemical Analysis

In PEG-MZF-NPs/CD44-shRNA/MFH mice group, tumor tissue and viscera tissue pathological specimens were subjected to HE staining, and the morphological changes in tissues were observed under a light microscope (BX46, Olympus, Japan). Tail vein blood was extracted from normal nude mice (no tumor, no treatment) and PEG-MZF-NPs/CD44-shRNA/MFH mice group after 6 weeks of treatment. The changes in serum alanine transaminase (ALT), aspartate aminotransferase (AST), and blood urea nitrogen (BUN), and Cr were detected before and after treatment to observe the effect of the combined treatment on liver and kidney function. In addition, the changes in white and red cells, hemoglobin, and platelets in blood were determined to detect the effect of combined therapy on bone marrow hematopoiesis.

### Statistical Analysis

Statistical analyses were performed using Excel or GraphPad Prism 8. All experiments were performed at least three times using a minimum of three replicates/condition in each experiment. Results of representative experiments are shown in the figures and tables. Statistics are reported as the mean ± standard deviation. Error bars represent standard deviation. Comparisons between two groups were performed using an unpaired Student’s t-test. Comparisons of >2 groups were performed using one-way ANOVA followed by Tukey’s *post hoc* test. *p*<0.05 was considered to indicate a statistically significant difference.

## Results

### Preparation and Physicochemicals of PEG-MZF-NPS/DDP Nanoliposomes

**Figure 2:**
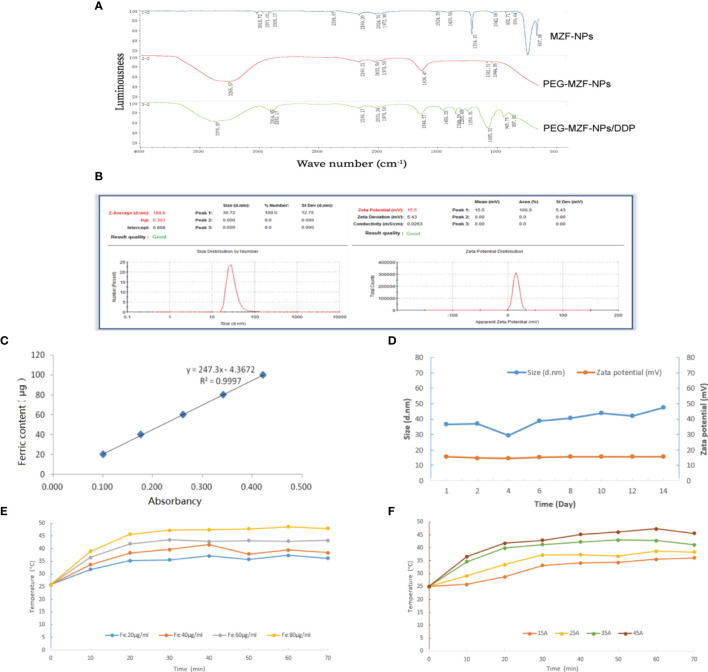
Characterization analysis of PEG-MZF-NPs/DDP nanoliposomes. **(A)** Infrared spectra of PEG-MZF-NPs and PEG-MZF-NPs/DDP nanoliposomes. **(B)** The hydrate particle size (left) and potential values (right) of PEG-MZF-NPs/DDP nanoliposomes. **(C)** The iron content of PEG-MZF-NPs/DDP nanoliposomes. **(D)** Stability curves of PEG-MZF-NPs/DDP nanoliposomes. **(E)** The heating curves of PEG-MZF-NPS/DDP nanoliposomes with different concentration in AMF. **(F)** The heating curves of PEG-MZF-NPS/DDP nanoliposomes in AMF with different output current.

The results of FTIR analysis indicated that the characteristic peaks of PEG on the surface of PEG-MZF-NPs/DDP nanoliposomes, including the Omurh stretching vibration at 3,376 cm^−1^, the symmetrical stretching vibration at 2,914 cm^−1^, and the bending vibrations at 1,645, 1,456, and 1,349 cm^−1^. The new peaks appeared near 945 and 887 cm−1, which were platinum (Pt) characteristic peaks, indicating that PEG had been successfully coated on the surface of MZF-NPs and that DDP had been encapsulated within (**Figure 2A**). As shown in **Figure 2B**, the hydrodynamic particle size detected by DLS was 30.72 ± 12.76 nm, polydispersity (PDI) was 0.303, and the potential values were 15.5 ± 5.43 mV. The iron content of PEG-MZF-NPs/DDP nanoliposomes was 0.52 mg/ml, as detected by o-phenanthroline spectrophotometry (**Figure 2C**). After 2 weeks of continuous PEG-MZF-NPS/DDP analysis at room temperature, the DLS slightly increased, and the potential showed a stable trend (**Figure 2D**). Of the tested concentrations, the magnetic fluid with 60 µg/ml rapidly warmed within 20 min, then gradually stabilized at 43°C or so (**Figure 2E**). Meanwhile, PEG-MZF-NPs/DDP nanoliposomes (Fe, 60 µg/ml) was exposed to a high-frequency alternating electromagnetics with 15, 25, 35, and 45 A for 60 min, respectively. As shown in **Figure 2F**, the temperature of the magnetic fluid at different electricity all rapidly increased within 20 min, then tended to be stable. As the output current increased, the maximum temperature rose. Of the tested output currents, the magnetic fluid with 35 A rapidly warmed within 20 min, then gradually stabilized at 43°C or so. This is the effective temperature range for tumor treatment and does not cause damage to normal tissue. A total of 60 µg/ml of Fe and 35 A of output current were thus selected as the concentration and the output current for magnetic fluid hyperthermia in the later experiments *in vitro* and *in vivo*.

### CD44 Gene Expression of Ovarian Cancer HO8910 Cells Transfected With CD44-shRNA Plasmids

**Figure 3:**
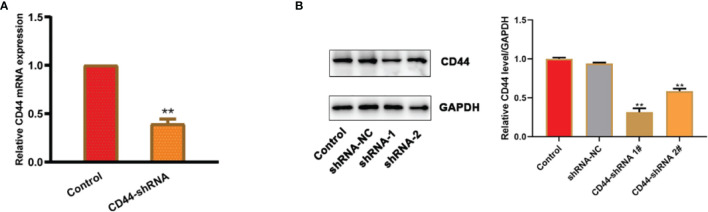
CD44 gene expression of ovarian cancer HO8910 cells transfected with CD44-shRNA plasmids. **(A)** CD44 mRNA detected by RT-PCR, **p < 0.01. **(B)** CD44 protein examined by Western blot (a, protein bands; b, quantitative statistical results according to the protein bands, **p < 0.01 vs. the untransfected cells).

The results of RT-PCR (**Figure 3A**) and Western blot (**Figure 3B**) experiments showed that the expression of CD44 mRNA and protein were both significantly decreased in comparison with the untransfected cells. Because shRNA-1 showed better inhibitory effects, it has been selected for further analysis.

### Construction of PEG-MZF-NPs/DDP/CD44-shRNA Nanoliposomes

**Figure 4:**
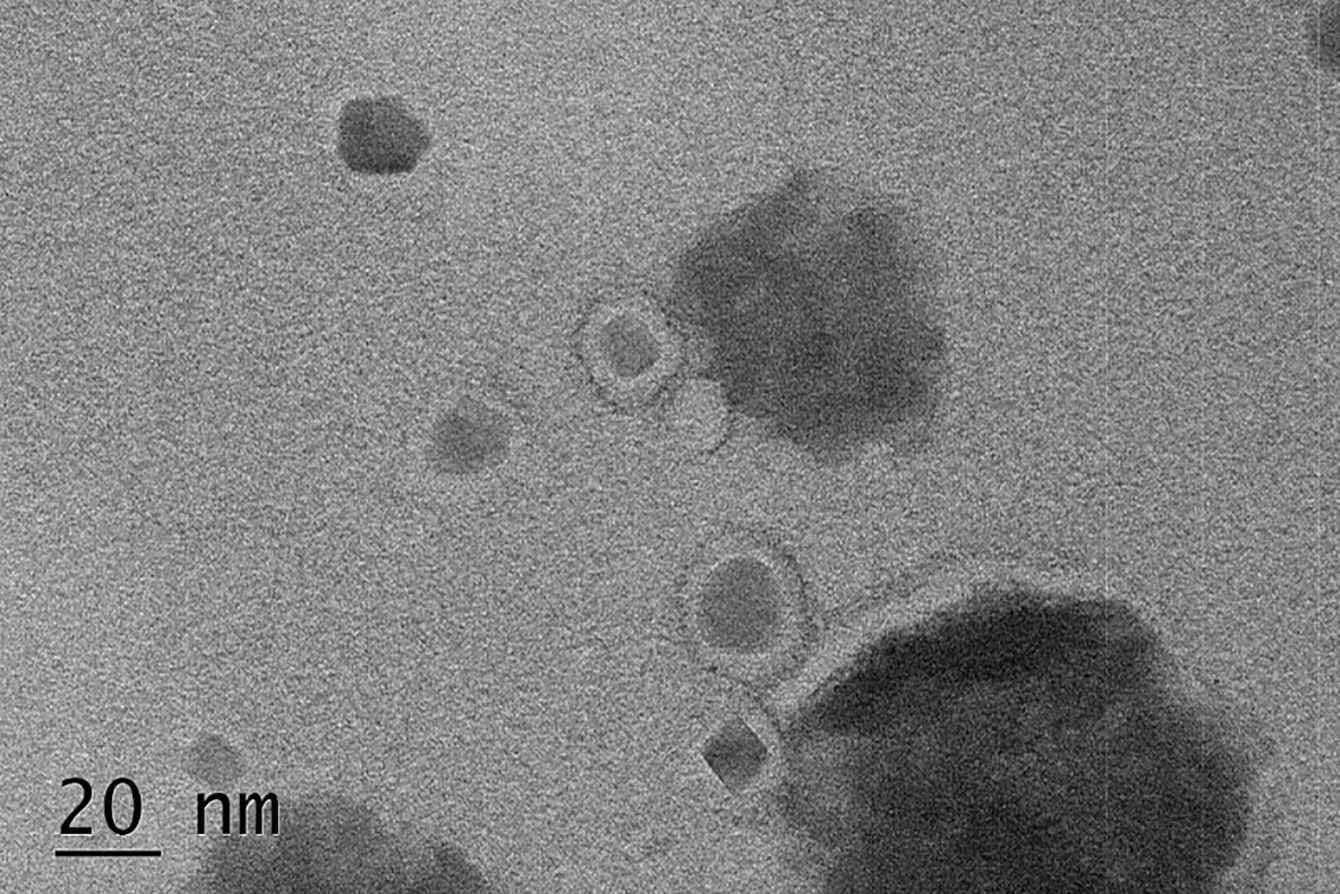
The negative stained PEG-MZF-NPs/DDP/CD44-shRNA nanoliposomes observed by TEM.

To complement each other’s advantages and create synergistic effects, we coupled CD44-shRNA to PEG-MZF-NPs/DDP and constructed PEG-MZF-NPs/DDP/CD44-shRNA nanoliposomes by thin film-ultrasonic method and high-speed stirring. TEM detection indicated that the nanoliposomes was 15–25 nm in diameter (**Figure 4**).

### The Effect of DDP/CD44-shRNA Nanoliposomes in AMF on Ovarian Cancer Cells *In Vitro*

**Figure 5:**
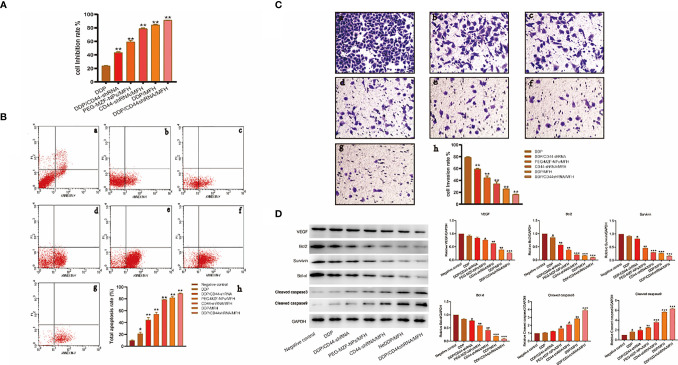
The *in vitro* therapeutical effects on ovarian cancer HO8910 cells intervened by the combined DDP chemotherapy, CD44-shRNA gene therapy, and MFH. **(A)** The cell proliferation inhibition rate of each group tested by MTT. **p < 0.01 vs. that of negative control group. **(B)** The cell apoptosis of each group analyzed by flow cytometry (a, negative control group; b, DDP group; c, DDP/CD44-shRNA group; d, PEG-MZF-NPs/MFH group; e, CD44-shRNA/MFH group; f, DDP/MFH group; g, DDP/CD44-shRNA/MFH group; h, quantitative statistical results). **(C)** Results of HO8910 cells’ invasion experiment in different experimental groups (a, negative control group; b, DDP group; c, DDP/CD44-shRNA group; d, PEG-MZF-NPs/MFH group; e, CD44-shRNA/MFH group; f, DDP/MFH group; g, DDP/CD44-shRNA/MFH group; h, quantitative statistical results). **(D)** VEGF, survivin, Bcl-2, Bcl-xl, cleaved caspase-3, and cleaved caspase-9 protein expression in different groups. *p < 0.05, ***p < 0.001 v.s. negative control.

As shown in **Figure 5**, the proliferation inhibition rate of ovarian cancer HO8910 cells treated with DDP/CD44-shRNA/MFH was (91.33 ± 0.22)%, significantly higher than (23.71 ± 0.54)% of DDP group, (43.47 ± 1.17)% of DDP/CD44-shRNA group, (59.34 ± 2.20)% of MFH group, (78.87 ± 1.06)% of CD44-shRNA/MFH group, and (84.32 ± 1.02)% of DDP/MFH group, *p*<0.05 (**Figure 5A**). Correspondingly, the apoptotic cell percentage of ovarian cancer HO8910 cells treated with DDP/CD44-shRNA/MFH was (89.12 ± 0.83)%, much higher than any of the other groups [negative control group: (9.89 ± 0.91)%; DDP group: (21.33 ± 2.22)%; DDP/CD44-shRNA group: (44.59 ± 3.93)%; MFH group: (54.13 ± 3.14)%; CD44-shRNA/MFH group: (78.88 ± 0.07)%; DDP/MFH group: (81.87 ± 1.93)%, *p*<0.05] **(** **Figure 5B****)**. Moreover, DDP/CD44-shRNA nanoliposomes in AMF significantly inhibited HO8910 cells’ invasiveness, far better than the other therapies did. The cell invasion rate of the DDP/CD44-shRNA/MFH group was only (16.60 ± 0.52)%, whereas that of the DDP group, DDP/CD44-shRNA group, MFH group, CD44-shRNA/MFH group, and DDP/MFH group reached (78.87 ± 1.30)%, (59.98 ± 2.40)%, (44.56 ± 2.89)%, (34.35 ± 3.44)%, and (25.51 ± 0.56)%, respectively (*p*<0.05), showing an obvious superiority of the combinatorial therapy (**Figure 5C**). Finally, as shown in **Figure 5D**, compared with the control, the protein expression of VEGF, survivin, Bcl-2, and Bcl-xl most significantly decreased in the DDP/CD44-shRNA/MFH group, specifically the expression of survivin and Bcl-2. The expression of cleaved caspase-3 and cleaved caspase-9 proteins was enervated in the negative control group and expressively increased in the DDP/CD44-shRNA/MFH group.

### *In vivo* Analysis of the PEG-MZF-NPs/DDP/CD44-shRNA Magnetic-Induced Heating for Ovarian Cancer Treatment

**Figure 6:**
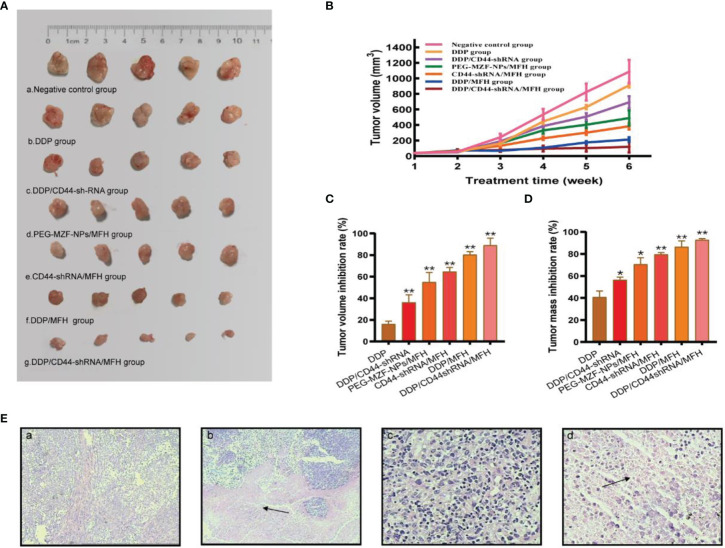
The *in vivo* therapeutic effects of the combined DDP chemotherapy, CD44-shRNA gene therapy, and MFH on nude mice bearing xenograft ovarian cancer. **(A)** Tumor appearance (a, negative control group; b, DDP group; c, DDP/CD44-shRNA group; d, MFH group; e, CD44-shRNA/MFH group; f, DDP/MFH group; g, DDP/CD44-shRNA/MFH group). **(B)** Tumor volume between the 6th week and the 1st week (*p < 0.05, **p < 0.01). **(C)** Tumor volume inhibition rate of tumor-bearing mice after treatment. **p < 0.01 vs. that of negative control group. **(D)** Tumor mass inhibition rate of tumor-bearing mice after treatment. *p < 0.05, **p < 0.01 vs. that of negative control group. **(E)** HE histopathological examination of ovarian cancer tissues. a, Negative control group (×100); b, DDP/CD44-shRNA/MFH group (100×); c, negative control group (×400); d, DDP/CD44-shRNA/MFH×400). The arrows in b and d represent necrotic focus.

There was a statistical difference in tumor size between the sixth week and the first week in tumor-bearing mice by different treatment methods (a, negative control group; b, DDP group; c, DDP/CD44-shRNA group; d, MFH group; e, CD44-shRNA/MFH group).The tumor mass and the volume inhibition rates of the DDP/CD44-shRNA/MFH group was (92.80 ± 1.09)% and (89.02 ± 6.68)%, respectively, far better than any of the other groups did [DDP group: (40.82 ± 5.66)% and (16.10 ± 2.79)%; DDP/CD44-shRNA group: (56.53 ± 2.40)% and (36.21 ± 6.99)%; MFH group: (70.63 ± 5.94)% and (54.95 ± 8.93)%; CD44-shRNA/MFH group: (79.59 ± 1.59)% and (64.67 ± 3.84)%; and DDP/MFH group: (86.49 ± 5.36)% and (80.35 ± 2.73)%, *p*<0.05] (**Figures 6A–D**). Histopathological examination showed that after magnetic-induced heating of the CD44-shRNA/DDP magnetic nanoliposomes, the ovarian tumor tissue presented a large red map-like necrotic focus, the tumor cells displayed coagulative necrosis, and the nucleus disintegrated and disappeared (**Figure 6E**).

**Figure 7:**
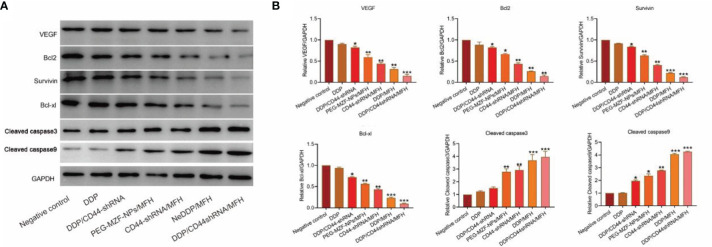
VEGF, survivin, Bcl-2, Bcl-xl, cleaved caspase-3, and cleaved caspase-9 protein expression of tumor tissues from nude mice bearing xenograft ovarian cancer in each treatment group tested by Western blot. **(A)** The protein bands. **(B)** Quantitative statistical results according to the protein bands. *p < 0.05, **p < 0.01 vs. that of negative control group. ***p < 0.001 v.s. negative control.

As shown in **Figure 7**, compared with the control group, the protein expression of VEGF, survivin, Bcl-2, and Bcl-xl decreased in each treatment group, but most significantly decreased in the DDP/CD44-shRNA/MFH group, specifically the expression of survivin and Bcl-2. The expression of cleaved caspase-3 and cleaved caspase-9 proteins was enervated in the negative control group but increased in all the treatment groups and expressively increased most in the DDP/CD44-shRNA/MFH group.

**Figure 8:**
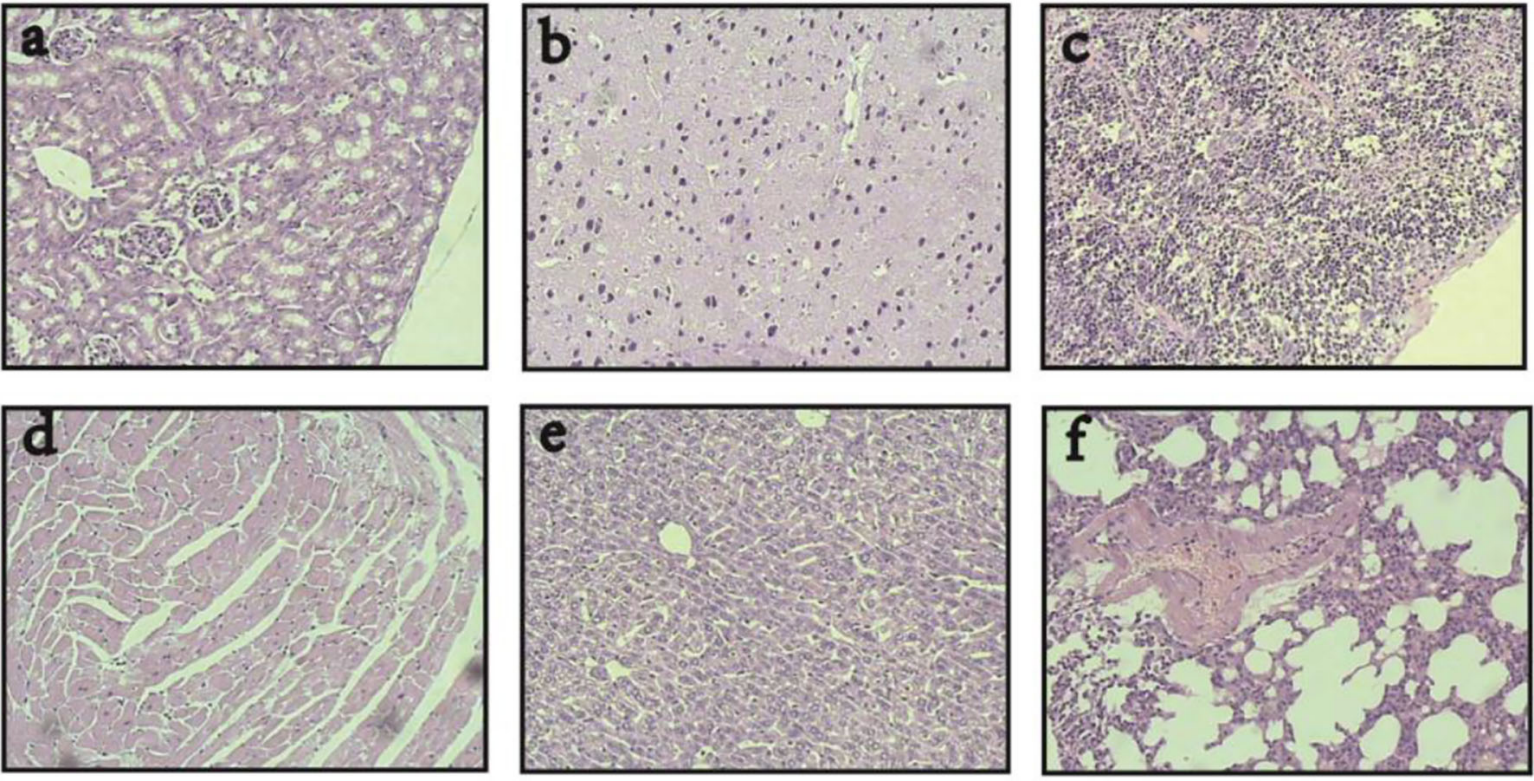
Visceras HE histopathological examination of nude mice bearing xenograft ovarian cancer treated with DDP/CD44-shRNA/MFH (400×). **(A)** kidney; **(B)** brain; **(C)** spleen; **(D)** heart; **(E)** liver; **(F)** lung.

In addition, the histopathological examination showed that after the combinatorial therapy, magnetic-induced heating of the CD44-shRNA/DDP nanoliposomes in this study selectively targeted ovarian cancer tissues and caused no damage to the visceras including the heart, liver, spleen, lung, kidney, brain, and pancreas (**Figure 8**).

In the blood routine tests, the white cell, red cell, Hb, and platelet of normal nude mice was (5.3 ± 0.7)×10^9^/L, (3.3 ± 0.4)×10^12^/L, (110 ± 7.9) g/L, and (303 ± 61)×10^9^/L, respectively. After treatment with CD44-shRNA/DDP magnetic nanoliposomes with MFH, the blood white cells of five nude mice bearing tumor were 5.9×10^9^/L,4.7×10^9^/L, 5.6×10^9^/L, 4.6×10^9^/L, and 5.8×10^9^/L, respectively; the red cells were 3.4×10^12^/L, 3.6×10^12^/L, 3.0×10^12^/L, 3.4×10^12^/L, and 3.5×10^12^/L,respectively; Hb was 115, 106, 105, 103, and 98 g/L, respectively; and the platelets were 362×10^9^/L, 306×10^9^/L, 307×10^9^/L, 256×10^9^/L, and 274×10^9^/L, respectively. In comparison with normal nude mice (without tumor and without treatment), the blood cells’ count and Hb concentration were all within the corresponding reference values, indicating that the combinational therapy had no significant adverse effect on bone marrow hematopoiesis. Similarly, in the serum biochemistry detection, ALT (U/L), AST (U/L), BUN (mmol/L), and Cr (µmol/L) of normal nude mice were 28.4 ± 4.4, 73.7 ± 12.2, 6.3 ± 0.8, and 53.7 ± 5.8, respectively. As for five nude mice bearing tumor in the combinational therapy group, ALT(U/L) was 31.5, 26.9, 27.9, 25.7, and 26.4, respectively; AST (U/L) was 83.3, 65.8,76.6, 85.5, and 73.8, respectively; BUN (mmol/L) was 5.8, 5.5, 5.9, 6.4, and 7.0, respectively; Cr (µmol/L) was 57.8, 58.3, 52.4, 50.7, and 56.3, respectively.

## Discussion

In the present study, we developed PEG-MZF-NPs/DDP/CD44-shRNA magnetic nanoliposomes and inaugurated an integrated therapy through the synergistic effect of MFH, gene therapy, and chemotherapy, and a satisfactory therapeutic effect on ovarian cancer *in vitro* and *in vivo* is shown.

Metal nanoparticles have broadly applicable prospects and recently have received extensive attention in the fields of medicine and pharmacy (16, 17). Currently, Manganese (Mn), an important metal in metabolism, attracts scientists’ attentiveness. Rehman et al. (18) prepared Mn0.5Zn0.5EuxDyxFe1.8–2xO4, selectively targeting cancer cells by the sonochemical method. PEG is one of the few polymers used in humans, also the most used magnetic nanoparticles’ surface modifier (19). It can bind to nanoparticles’ surface through end-group reactivity and bind to the DNA fragments, drugs, and other biological fragments (20, 21). Owing to its good water solubility, biocompatibility, and stability, PEG is highly favored to be used for modifying organic and inorganic nanomaterials for drug delivery or gene transferring (22). In our previous study, we synthesized MZF-NPs and coated them with PEG (PEG-MZF-NPs) and confirmed that MZF-NPs and PEG-MZF-NPs both have good magnetic-heat effects and the temperature could be maintained 43°C or so in AMF by controlling the nanoparticles’ concentration or adjusting magnetic field intensity (23). Interestingly, this temperature scope is extremely applicable for tumor thermotherapy. When PEG-MZF-NPs were used as magnetic media for tumor hyperthermia, they showed good effects (15). In the present study, PEG-MZF-NPs were loaded with DDP, and PEG-MZF-NPs/DDP nanoliposomes were prepared by the impregnation method. After being stored at 4°C for a week, a small amount of brown precipitate was seen at the bottom of the tube, and the solution was slightly layered but could be re-suspended after ultrasonic dispersion, indicating that it had good suspensibility. Studies have shown that if the tumor’s temperature reaches 41–47°C, this directly damages cancer cells and prevents DNA repair and promotes cell death (24). Magnetic nanoparticles-induced hyperthermia has attracted great attention in tumor therapy due to its good heating response under the application of AMF, which can induce cancer cell death through the MFH. Some studies have shown that nanoparticles are the most stable when the surface charge is −30–30 mV (25). In this study, we confirmed that 60 µg/ml of Fe and 35 A of output current is the effective temperature range for tumor treatment and does not cause damage to normal tissue, which was similar to PEG-MZF-NPs (26).

Adhesion molecule CD44, highly expressed in ovarian cancer tissue, plays a crucial role in tumor occurrence and development and is also related to cancer chemo-resistance (27). It has been evidenced that CD44 gene knockout can inhibit tumor growth, invasion, and metastasis and also can increase the sensibility of chemotherapeutics (28). In one of our previous studies, we constructed CD44-shRNA plasmids and transfected them into ovarian cancer SKOV-3 cells by PEG-MZF-NPs; CD44 gene expression was significantly inhibited, indicating CD44-shRNA has a good potential to treat ovarian cancer (15). In this study, ovarian cancer HO8910 cells were transfected with CD44-shRNA plasmids by using PEG-MZF-NPs as gene transferring vector. In present study, we confirmed that the mRNA and protein expression of CD44 were both significantly decreased in comparison with the untransfected cells, which indicated that CD44 gene may be a candidate therapeutic target for ovarian cancer, and using PEG-MZF-NPs to transfer objective gene in gene therapy is workable.

Honestly, chemotherapy, gene therapy, and thermotherapy all play important roles in cancer treatment. However, they each has their own merits and demerits. It is difficult to completely eliminate tumor in any of the individuals. To complement each other’s advantages and create synergistic effects, we coupled CD44-shRNA to PEG-MZF-NPs/DDP and constructed PEG-MZF-NPs/DDP/CD44-shRNA nanoliposomes by thin film-ultrasonic method and high-speed stirring. Jordan et al. (29) reported that in tumor cells bearing magnetic particles, the external magnetic field could cause these particles to heat, promoting tumor cell death, and MFH was thus born as a result. Hyperthermia can not only kill tumor cells directly by raising temperature but also play a complementary and synergistic effect with radiotherapy, chemotherapy, and immunotherapy (30). Furthermore, hyperthermia can improve the sensitivity of chemotherapeutic drugs (11). The current study also found that the combined DDP chemotherapy, CD44-shRNA gene therapy, and MFH could inhibit HO8910 cells growth and induce cells’ apoptosis, and the curative effects were better than that of any single therapy.

In order to further explore the specific role of PEG-MZF-NPs/CD44-shRNA/DDP nanoliposomes, we established a human ovarian cancer xenograft model in nude mice. A previous study found that taking advantage of Mn0.5Zn0.5Fe2O4 temperature control ability combined with As2O3 chemotherapy has a significant effect on liver cancer treatment (31). Injecting experimental animals bearing tumors with a solution containing magnetic nanoparticles and then subjecting them to a magnetic field to increase the magnetic particles’ temperature resulted in a significant reduction in tumor growth (32). Xie et al. (10) prepared magnetic Mn–Zn ferrite nanocrystals coated with PEG and injected them into the mice’s tail vein for targeted therapy. Subjecting the tumor tissues to a magnetic field *in vitro* raised their temperature to 43°C and promoted tumor inhibition. Combining chemotherapy, magnetic hyperthermia, and gene therapy can improve tumor treatment’s efficiency, reduce drug dosage, and lower adverse reactions (28). Our *in vitro* and *in vivo* analysis, the WB results suggested that CD44-shRNA/DDP/MFH might promote cell apoptosis by downregulating the expression of survivin, Bcl-2, and Bcl-xl protein and upregulating the expression of caspase-3 and caspase-9 protein (which were all known as proliferation or apoptosis biomarkers); also, it inhibits tumor neovascularization by inhibiting the expression of VEGF protein, thus inhibiting ovarian cancer tumor growth, invasion, and migration. Therefore, we suggest that CD44-shRNA/DDP magnetic nanoliposomes have a selectivity to ovarian cancer tissues and cells and may be a potential candidate for ovarian cancer therapy.

The current study has limitations. The results suggested that the proliferation inhibition and apoptosis rate of ovarian cancer HO8910 cells treated with DDP/CD44-shRNA/MFH was 91.33 ± 0.22%, while that of the DDP/MFH group was 84.32 ± 1.02%, indicating that the inhibition is mainly due to the chemotherapeutic drug and MFH with little effect of combined CD44-shRNA. In future study, we will separate DDP, CD44-SHRNA, and MFH into separate groups in the future study to further clarify the therapeutic efficacy of single treatment regimen for ovarian cancer.

## Conclusion

In the present study, we developed PEG-MZF-NPs/DDP/CD44-shRNA magnetic nanoliposomes to inaugurate combined therapy through the synergistic effect of magnetic fluid hyperthermia, gene therapy, and chemotherapy. The *in vitro* and *in vivo* results showed that the therapeutic effect of this combination regimen on ovarian cancer was much better than that of any single therapy, with no adverse effects. Magnetic-induced-heating PEG-MZF-NPs/DDP/CD44-shRNA nanoliposomes provide a new and feasible method for ovarian cancer treatment.

## Data Availability Statement

The original contributions presented in the study are included in the article/supplementary material. Further inquiries can be directed to the corresponding author.

## Author Contributions

TG conception, writer, and design. YM materials and data collection. YZ analysis and interpretation. FW funding and critical review. ZL literature review and supervision. ML corresponding author, submission. All authors contributed to the article and approved the submitted version.

## Funding

This work was financially supported by the National Natural Science Foundation of China (81571797), the Natural Science Foundation of Nanjing University of Chinese Medicine China (XZR2020093), the Social Development Plan of Taizhou, China (TN202108),and Taizhou People’s Hospital Medical Innovation Team Foundation, China (CXTDA201901), and the project of Taizhou People’s Hospital (ZL202023).

## Conflict of Interest

The authors declare that the research was conducted in the absence of any commercial or financial relationships that could be construed as a potential conflict of interest.

## Publisher’s Note

All claims expressed in this article are solely those of the authors and do not necessarily represent those of their affiliated organizations, or those of the publisher, the editors and the reviewers. Any product that may be evaluated in this article, or claim that may be made by its manufacturer, is not guaranteed or endorsed by the publisher.
